# Neurological Predictors of Clinical Outcomes in Hospitalized Patients With COVID-19

**DOI:** 10.3389/fneur.2020.585944

**Published:** 2020-10-30

**Authors:** Hisham Salahuddin, Ehad Afreen, Irfan S. Sheikh, Sohaib Lateef, Giana Dawod, Judy Daboul, Nurose Karim, Khaled Gharaibeh, Mustafa Al-Chalabi, Sihyeong Park, Alicia C. Castonguay, Ragheb Assaly, Fadi Safi, Marla Matal, Ajaz Sheikh, Gretchen Tietjen, Deepa Malaiyandi, Elysia James, Imran Ali, Syed F. Zaidi, Ahmad Abdelwahed, Vieh Kung, Richard Burgess, Mouhammad A. Jumaa

**Affiliations:** ^1^ProMedica Neurosciences Center, Toledo, OH, United States; ^2^Department of Neurology, College of Medicine and Life Sciences University of Toledo, Toledo, OH, United States; ^3^St. Luke's Hospital, Maumee, OH, United States; ^4^Department of Internal Medicine, College of Medicine and Life Sciences, University of Toledo, Toledo, OH, United States; ^5^Anesthesiology and Critical Medicine, ProMedica Physicians, Toledo, OH, United States; ^6^Pulmonary Medicine, Critical Care Medicine, Sleep Medicine, ProMedica Physicians, Toledo, OH, United States

**Keywords:** neurological, COVID-19, coronavirus, mortality, encephalopathy, stroke

## Abstract

**Introduction:** Multiple risk factors of mortality have been identified in patients with COVID-19. Here, we sought to determine the effect of a history of neurological disorder and development of neurological manifestations on mortality in hospitalized patients with COVID-19.

**Methods:** From March 20 to May 20, 2020, hospitalized patients with laboratory confirmed or highly suspected COVID-19 were identified at four hospitals in Ohio. Previous history of neurological disease was classified by severity (major or minor). Neurological manifestations during disease course were also grouped into major and minor manifestations. Encephalopathy, ischemic or hemorrhagic stroke, and seizures were defined as major manifestations, whereas minor neurological manifestations included headache, anosmia, dysgeusia, dizziness or vertigo, and myalgias. Multivariate logistic regression models were used to determine significant predictors of mortality in patients with COVID-19 infection.

**Results:** 574/626 hospitalized patients were eligible for inclusion. Mean age of the 574 patients included in the analysis was 62.8 (SD 17.6), with 298 (51.9%) women. Of the cohort, 240(41.8%) patients had a prior history of neurological disease (HND), of which 204 (35.5%) had a major history of neurological disease (HND). Mortality rates were higher in patients with a major HND (30.9 vs. 15.4%; *p* = 0.00002), although this was not a significant predictor of death. Major neurological manifestations were recorded in 203/574 (35.4%) patients during disease course. The mortality rate in patients who had major neurological manifestations was 37.4% compared to 11.9% (*p* = 2 × 10^−12^) in those who did not. In multivariate analysis, major neurological manifestation (OR 2.1, CI 1.3-3.4; *p* = 0.002) was a predictor of death.

**Conclusions:** In this retrospective study, history of pre-existing neurological disease in hospitalized COVID-19 patients did not impact mortality; however, development of major neurological manifestations during disease course was found to be an independent predictor of death. Larger studies are needed to validate our findings.

## Introduction

Severe acute respiratory syndrome coronavirus 2(SARS-CoV-2), causative agent of the coronavirus disease 2019 (COVID-19) pandemic, is one of seven coronaviruses known to infect humans. The COVID-19 pandemic has become the most challenging public health crisis in decades. High mortality rate has been noted in elderly patients, patients with underlying medical risk factors, and nursing home residents. Along with age, chronic cardiac disease, and chronic pulmonary disease, a prior history of cerebrovascular disease was among the predictors of death in patients with COVID-19 (1, 2). However, limited data exists on the outcome of COVID-19 patients with other underlying neurological diseases.

Fever and cough are the most common symptoms of COVID-19, but disease presentations and clinical course are widely variable (3). Similar to severe acute respiratory syndrome coronavirus(SARS-CoV) and Middle East respiratory syndrome coronavirus(MERS-CoV), SARS-CoV-2 is hypothesized to have neurotropic properties (4). Many neurological manifestations were previously reported during outbreaks of SARS-CoV in 2003 and the MERS-CoV in 2012 (5). Although SARS-CoV-2 is primarily a respiratory virus, a growing body of literature has highlighted a high incidence of neurological manifestations in hospitalized patients, which range from nonspecific symptoms, such as headache, dizziness, and myalgias to more severe complications including encephalopathy, cerebrovascular diseases, and myositis (6–8). An alarming incidence of neurological emergencies as early COVID-19 manifestations have also been reported (9, 10). The outcome of COVID-19 patients with neurological manifestations during the disease course remains unclear.

In this study, we evaluate the outcome of hospitalized COVID-19 patients with underlying neurological diseases and those who suffer from neurological manifestations during disease course.

## Methods

The study was approved by Institutional Review Boards at the four participating hospitals. Informed consent was waived due to the retrospective, expedited nature of the study. We retrospectively reviewed medical records of all hospitalized patients treated for COVID-19 at four regional hospitals, one of which was a designated COVID-19 hospital, between the 20th of March and May 20th, 2020. Patients were included in the analysis if they had a confirmed COVID-19 test by polymerase chain reaction (PCR) from nasopharyngeal swab or were determined to be highly suspected for COVID-19 infection with consistent CT chest imaging, and no alternative explanation for presenting symptoms as assessed by a fellowship trained infectious disease specialist.

Epidemiological, demographic, clinical, laboratory, treatment, and outcome data were collected from review of electronic medical records (HS, EA, ISS, SL, NK, MA, KG, PS, GD, JD) using a standardized data collection form. The data collection form focused on history of neurological disease, neurological manifestations at presentation and while in hospital. Data was compiled by one of the authors (HS) and data was checked for discrepancies by two of the authors (HS, MJ).

### Neurological Disease and Manifestations

History of pre-existing neurological disease (HND) was grouped into major or minor disease. History of major neurological disease included dementia, ischemic or hemorrhagic stroke, epilepsy, traumatic brain injury, multiple sclerosis, Parkinson's disease, and developmental delay. Neurological manifestations were grouped according to whether patient presented to the hospital with early neurological manifestations (ENM) and/or developed neurological manifestations while in hospital (NMH). We also grouped neurological manifestations into major and minor categories. We defined Major ENM and NMH to include encephalopathy, ischemic or hemorrhagic stroke, and seizures. Minor ENM and NMH included but were not limited to dizziness or vertigo, anosmia, dysgeusia, headache, and myalgia. As myalgias may also be considered a non-neurological manifestation, we also recorded the number of patients who developed myalgia as the only neurological manifestation.

### Statistical Analysis

The data set was compiled in Microsoft Excel and exported to a statistical analysis software R: A language and environment for statistical computing; EZR version 1.32 (Saitama Medical Center, Jichi Medical University, Saitama, Japan). Continuous and categorical variables were presented as mean (standard deviation) and median (interquartile range). Univariate analysis was performed to find significant factors associated with mortality. To limit overfitting the multivariate analysis, we used the strongest univariate predictors from past medical history and presenting symptoms.

## Results

### Study Population

**Figure 1:**
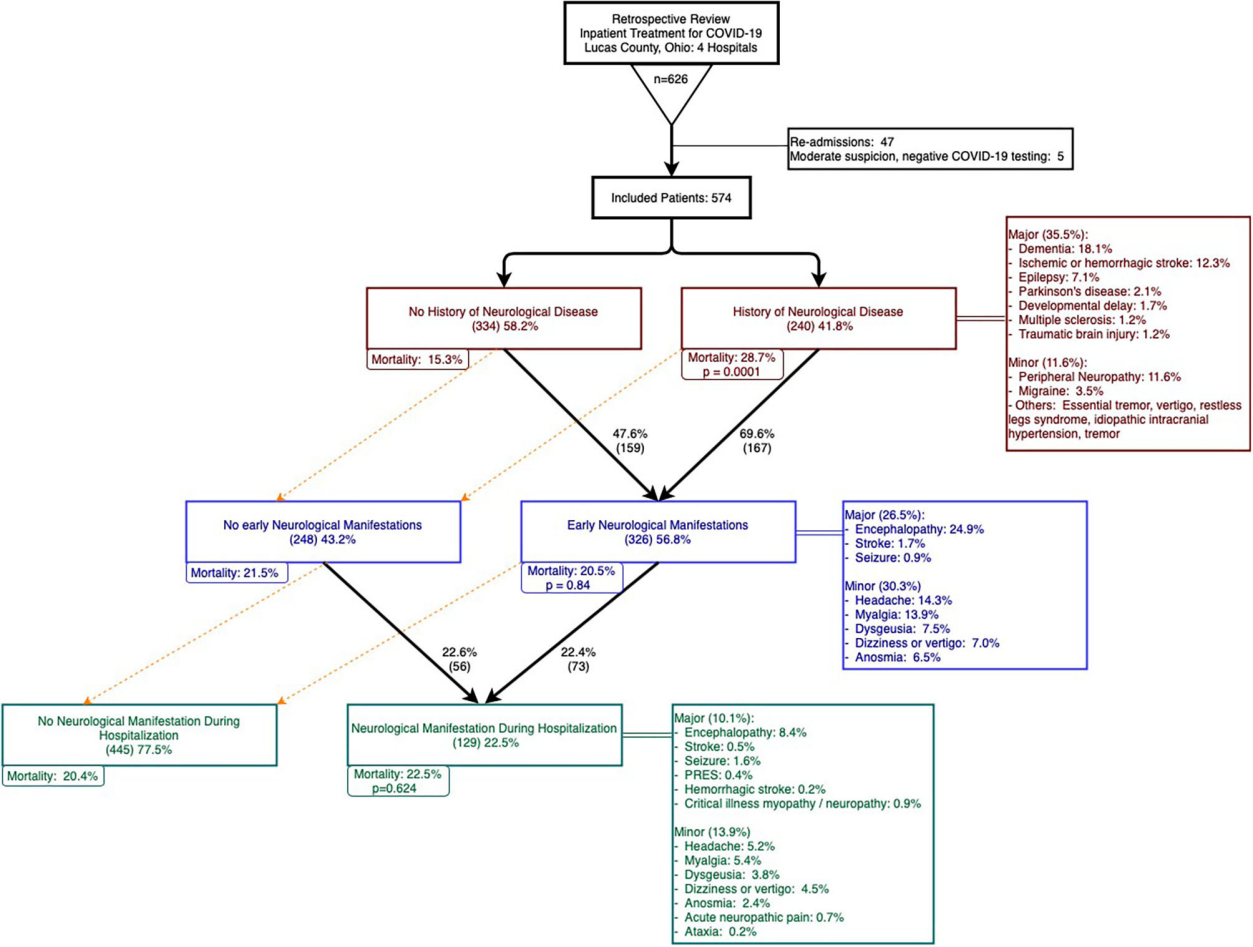
Patient selection, history of neurological disease (HND), early neurological manifestations (ENM), and neurological manifestations in hospital (NMH).

Over a two-month period between March 20–May 20, 2020, 626 patients were admitted and treated for COVID-19 in four hospitals in Lucas County, Ohio. After excluding re-admissions and patients with negative COVID tests with moderate suspicion of COVID-19, 574 patients were included in our analysis. Among these patients, 562 patients (97.9%) had a positive SARS-CoV-2 PCR test and two patients had positive SARS-CoV-2 antibodies. The remaining 10 patients had negative SARS-CoV-2 tests but were deemed to be highly suspicious as determined by infectious disease experts. Figure 1 summarizes patient selection in the study.

### Demographics and Clinical Characteristics

Baseline demographics and co-morbidities are summarized in Table 1. Mean age of the cohort was 62.8 ± 17.6 years, with the youngest and oldest patients being 3 months and 98 years old respectively, and patients over the age of 55 years constituted 71.3% of the cohort. Approximately one fifth of the population presented from an extended care facility (ECF) (19.9%), and 384 (66.9%) of patients presented from home. The remaining patients presented from assisted living facilities (12.7%) or a correctional facility (0.5%). Most patients were Caucasian (57.1%) or African American (38%). Mean body mass index was 32.9 (SD 13.3) and 9% of patients were healthcare workers.

**Table 1:** Baseline demographics of COVID-19 cohort.

	**Participants *n* = 574 (%)**	**Mean**	**SD**
Age		62.83	17.55
Gender			
Male	276 (48.1)		
Female	298 (51.9)		
Ethnicity			
White	328 (57.1)		
African American	218 (38.0)		
Asian	5 (0.9)		
Hispanic	19 (3.3)		
Other	4 (0.7)		
BMI		32.9	13.3
Presentation from			
Home	384 (66.9)		
Assisted living	73 (12.7)		
ECF	114 (19.9)		
Correctional facility	3 (0.5)		
Healthcare workers	52 (9.1)		
Past medical history			
Hypertension	408 (71.1)		
DM	227 (39.6)		
Arrhythmia	97 (16.9)		
Asthma	77 (13.4)		
CAD	115 (20.0)		
CHF	91 (15.9)		
Cirrhosis	7 (1.2)		
CKD	127 (22.1)		
COPD	98 (17.0)		
Hyperlipidemia	293 (51.1)		
Malignancy	71 (12.4)		
Active	12 (2.1)		
History of malignancy	59 (10.3)		
OSA	91 (15.9)		
Peptic ulcer disease	18 (3.1)		
Mental health disorder	229 (39.9)		
Depression	155 (27)		
Anxiety	97 (16.9)		
Bipolar disorder	31 (5.4)		
Schizophrenia/Schizoaffective	32 (5.6)		
Other	19 (3.3)		

*BMI, body mass index; ECF, extended care facility; DM, diabetes mellitus; CAD, coronary artery disease; CHF, congestive heart failure; CKD, chronic kidney disease; COPD, chronic obstructive pulmonary disease; OSA, obstructive sleep apnea*.

The majority of patients (80.7%) had at least two or more comorbidities. The most common comorbidities were hypertension (71.1%), hyperlipidemia (51.1%), mental health disorders (39.9%), diabetes mellitus (39.6%), chronic kidney disease (22.1%), and coronary artery disease (20%). Common symptoms at presentation included shortness of breath or hypoxia (79.8%), fever (64.5%), cough (69.7%), and fatigue (45.5%). According to the WHO classification for COVID-19 (11), 60.8, 25.8, and 13.4% of the cohort had moderate, severe, or critical disease severity, respectively. In hospital medical complications included acute kidney injury (39.4%), sepsis or septic shock (27.9%), cardiac arrhythmias (19.9%), acute respiratory distress syndrome (17.4%), and cardiac injury (16.2%). Details of presenting illness and admission laboratory values are summarized in Supplementary Tables 1, 2.

Mean length of stay was 10.2 (SD 8.5) days with 30.5% of the cohort requiring intensive care unit (ICU) level of care and an average of 4.5 (SD 8.8) days in the ICU. Approximately one fifth (22.1%) of the study group required mechanical ventilation and half of all patients were placed in prone position as part of their treatment. Re-admission during the two-month period for worsening COVID symptoms or disease complications occurred in 5.7% of patients. Supplementary Tables 3, 4 summarizes hospital course, death rate, and reasons for re-admission.

Upon discharge, all patients who were admitted from an extended care facility (ECF) were discharged back to an ECF. An additional nine patients were discharged to an ECF, 5 patients to an inpatient rehabilitation facility, and the remaining 326 patients (56.8%) were discharged back home, to an assisted living facility, or correctional facility. One fifth of the population expired in the hospital or were discharged to hospice care (120; 20.9%). Death rates in patients between 45 and 54 and above 85 years of age were 1.2 and 44.6%, respectively. Supplementary Figure 1 summarizes death rates by age group. In univariate and multivariate analyses, age was the strongest predictor of death in our cohort.

### History of Neurological Disease

Our study focused on patients with a history of neurological disease (HND) and those who developed neurological manifestations with COVID-19. Patients with a history of any neurological disease comprised of 41.8% of the cohort and 35.5% had a history of a major neurological disease (Figure 1, HNDs are summarized in Table 2). Major HNDs included a history of dementia (18.1%), ischemic or hemorrhagic stroke (12.3%), epilepsy (7.1%), transient ischemic attack (TIA) (3.7%), Parkinson's disease (2.1%), developmental delay (1.9%), multiple sclerosis (1.2%), or traumatic brain injury (1.2%). Patients with any HND (28.7 vs. 15.3%; *p* = 0.0001) or a major HND (30.9 vs 15.4%; *p* = 0.00002) were more likely to expire.

**Table 2:** History of pre-existing neurological problems.

	**Participants *n* = 574 (%)**
History of neurological problems	**240 (41.8)**
Major CNS problem	**204 (35.5)**
Dementia	104 (18.1)
Stroke (ischemic or hemorrhagic)	71 (12.4)
Transient ischemic attack	21 (3.7)
Epilepsy	41 (7.1)
Parkinson's disease	12 (2.1)
Developmental delay	11 (1.9)
Multiple sclerosis	7 (1.2)
Traumatic brain injury	7 (1.2)
Minor CNS problem	**36 (6.27)**

*CNS, central nervous system. Bolding signifies that the number is part of the whole category*.

### Neurological Manifestations

Approximately two thirds of the cohort developed neurological manifestations during the course of COVID-19. Forty four percent of patients presented to the hospital with Early Neurological Manifestations (ENM), 9.8% developed Neurological Manifestations during Hospitalization (NMH), and 12.7% of the cohort had both ENM and NMH (see Table 3).

**Table 3:** Neurological symptoms at presentation and during hospital encounter.

	**Participants**
	***n* = 574 (%)**
**Early neurological manifestations (ENM)**	**326 (56.8)**
Major neurological symptoms	**152 (26.5)**
Encephalopathy	143 (24.9)
Seizure	5 (0.9)
Stroke	7 (1.2)
Minor neurological symptoms	**174 (30.3)**
Headache	82 (14.3)
Myalgia	80 (13.9)
Anosmia	37 (6.5)
Dizziness or vertigo	40 (7.0)
Dysgeusia	43 (7.5)
**Neurological manifestation while in hospital (NMD)**	**129 (22.5)**
Major in-hospital neurological complications	**58 (10.1)**
Encephalopathy	48 (8.4)
Seizure	9 (1.6)
Ischemic stroke	3 (0.5)
Critical illness myopathy/neuropathy	5 (0.9)
Posterior reversible encephalopathy syndrome (PRES)	2 (0.4)
Cerebral venous sinus thrombosis	1 (0.2)
Minor in-hospital neurological complications	**80 (13.9)**
Myalgia	31 (5.4)
Headache	30 (5.2)
Dizziness	26 (4.5)
Dysgeusia	22 (3.8)
Anosmia	14 (2.4)
Acute neuropathic pain	4 (0.7)
Ataxia	1 (0.2)
Fatigue	64 (11.2)

Patients with neurological manifestations (either at presentation or during their hospital stay) had significantly higher rates of ICU admission (33.5 vs. 22.4%; *p* = 0.007), longer mean ICU stays (8.3 ± 11.6 vs. 2.2 ± 5.3 days; *p* = 0.00003), longer overall hospital stays (14.1 ± 11.6 vs. 8.1 ± 5.2 days, *p* = 0.06), and required more days on mechanical ventilation (7 ± 10.9 vs. 1.6 ± 4.8 days; *p* = 0.001) (Supplementary Table 5).

**Figure 2:**
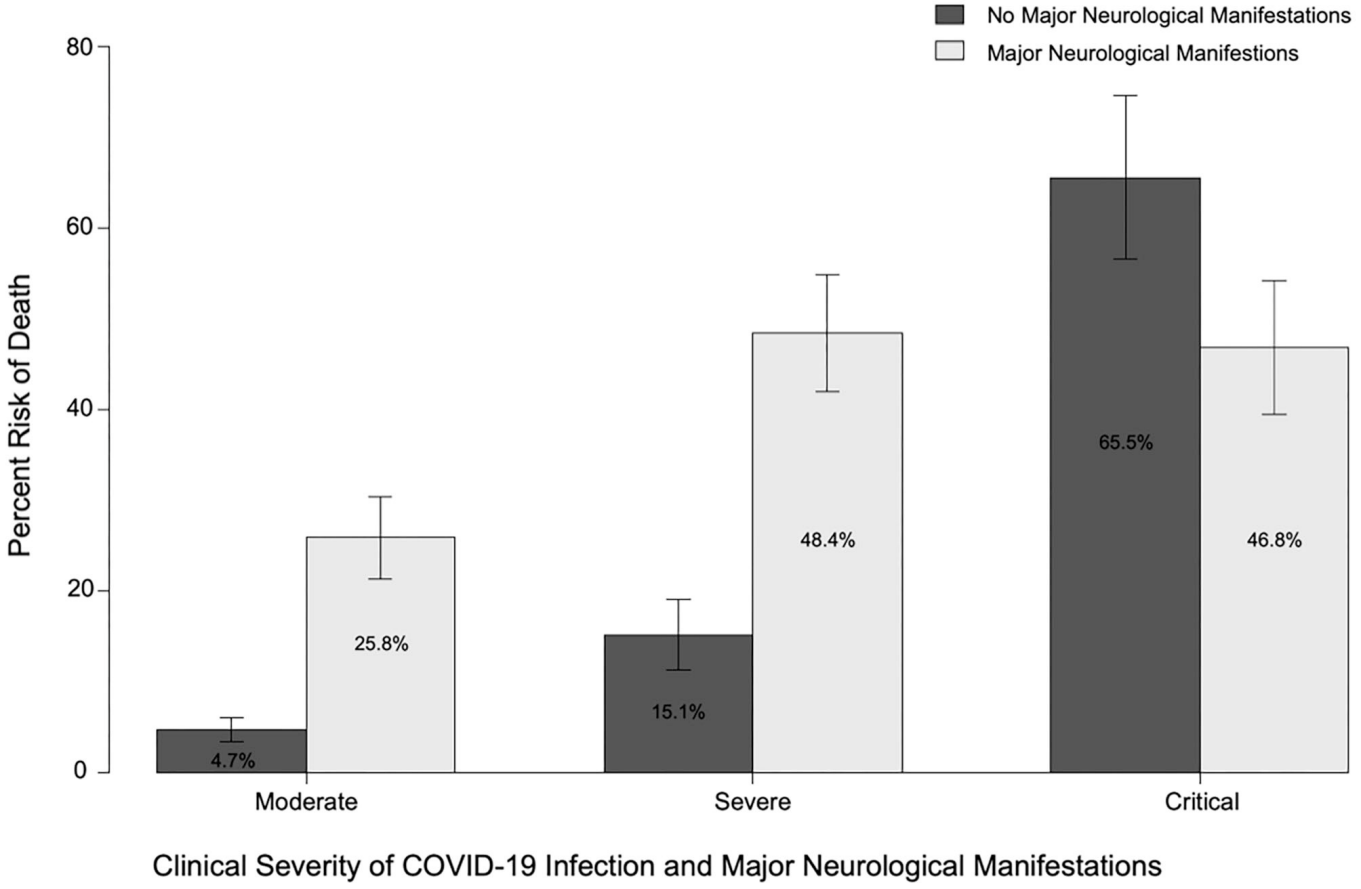
Risk of death in patients with or without major neurological manifestations (at any time) and moderate, severe, or critical COVID-19 disease.

Major neurological manifestations were present in 45.8, 30.5, and 23.6% of patients with moderate, severe, and critical COVID-19, respectively. (See Figure 2 for mortality by neurological manifestations and COVID-19 severity.) Major neurological manifestations were recorded in 203 (35.4%) patients, with 145 (25.3%) patients with ENM, 51 (8.9%) patients with NMH, and 7 (1.2%) patients with both ENM and NMH. Mortality was higher in patients with a major ENM (36.2 vs. 15.4%; *p* = 0.0000002) or major NMH (41.4 vs. 18.6%; *p* = 0.00024). Similarly, patients who developed a major neurological manifestation at any time during infection with COVID-19 had higher rates of mortality than those without any major neurological manifestations (37.4 vs. 11.9%; *p* = 2 × 10^−12^).

### Early Neurological Manifestations (ENM)

Of 326 patients with Early Neurological Manifestations (ENM), 152 (26.5%) patients had major and 174 had minor neurological symptoms. Encephalopathy was the most common major ENM (24.9%), followed by stroke (1.2%), and seizure (0.9%). Amongst minor neurological symptoms, headache (14.3%) was most common, followed by myalgia (13.9), dysgeusia (7.5%), dizziness or vertigo (7%), and anosmia (6.5%). Presentation with myalgia only as a minor neurological condition occurred in 47 patients (8%).

### Neurological Manifestations During Hospitalization (NMH)

Neurological Manifestations during Hospitalization (NMH) occurred in 129 (22.5%) patients. Median duration to development of neurological symptoms in-hospital was 3 (IQR 1–7) days. Development of major NMH occurred in 58 (10.1%) of patients, of which 7 patients had major ENM as well. Encephalopathy was the most common major NMH (8.4%), followed by seizure (1.6%), critical illness myopathy or neuropathy (0.9%), and ischemic stroke (0.5%). Minor NMH occurred in 13.9% of patients, with myalgia (5.4%), headache (5.2%), dizziness (4.5%), dysgeusia (3.8%), and anosmia (2.4%) the most common.

Uncommon neurological disorders with concurrent COVID-19 in our population included two patients with posterior reversible encephalopathy syndrome, one patient with cerebral venous sinus thrombosis, and one patient with a posterior inferior cerebellar artery pseudoaneurysm resulting in subarachnoid hemorrhage.

### Predictors of Mortality

After adjustment for gender, hypertension, disease severity on presentation, cardiac injury, ferritin, neutrophil, and lymphocyte counts, multivariate analysis revealed that age (OR 1.05; CI 1.03-1.07; *p* = 2.3 × 10^−8^), major neurological manifestation at any time (OR 2.1; CI 1.31-3.4; *p* = 0.0022), chronic kidney disease (OR 1.81; CI 1.1-2.95; *p* = 0.018), diarrhea on presentation (OR 0.53; CI 0.29-0.97; *p* = 0.041), heart failure (OR 9.5; CI 1.71-52.9; *p* = 0.01), and active or history of malignancy (OR 1.75; CI 1.09-2.81; *p* = 0.021) were predictors of death.

## Discussion

This retrospective cohort study of hospitalized patients with COVID-19 identified “major neurological manifestations” during disease course as an independent predictor of death. This finding can add a prognostic value to the care of this patient population. Encephalopathy was the most common ENM in our cohort (24.9%), with additional 8.4% developing encephalopathy during hospital stay. Impaired consciousness is a relatively common symptom of COVID-19 infection, and was reported in 37% of hospitalized patients in a cohort from Wuhan (6). Results from the ALBACOVID registry from Spain documented disorders of consciousness in 19.6% of infected patients, mostly in the severe infection group. Disorders of consciousness were associated strongly with older age, higher CK levels, lymphopenia, and advanced stages of COVID-19 (12). Encephalopathy presents with typical hallmark symptoms of fluctuating attention and arousal, with variable degrees of impairment in consciousness. Factors contributing to encephalopathy in COVID-19 patients include among many, hypoxia, metabolic abnormalities, cytokine storm, renal dysfunction, and sepsis (13).

Although meningoencephalitis could theoretically represent another explanation for encephalopathy, and SARS-CoV-1 and MERS-CoV are known to invade the central nervous system, such evidence is not yet clear in SARS-CoV-2. Moriguchi et al. reported a 24 year old patient with COVID-19 presenting with symptoms of altered mental status, fever, headache and seizures, who was confirmed (with MRI and cerebrospinal fluid (CSF) PCR) to have right temporal lateral-ventriculitis (14). In another report, a patient with encephalopathy was confirmed radiologically to exhibit bilateral thalamic, medial temporal, and sub-insular ring enhancing lesions (diagnosed with acute hemorrhagic necrotizing encephalopathy) (14). Two patients in our cohort underwent PCR testing in (CSF), both were negative.

Ischemic stroke occurred in 1.7% of patients in our cohort and all but one presented outside the time window or did not meet criteria for thrombolysis and/ or mechanical thrombectomy. One patient underwent mechanical thrombectomy and subsequently died from disease complications. The stroke incidence in our cohort is consistent with other recent studies (6, 15), despite the older median age and higher percentage of nursing home patients noted in our cohort. Ischemic stroke may occur due to concurrent risk factors (such as atrial fibrillation in patients with mild or asymptomatic COVID-19), as a complication of severe COVID-19 pathology (such as hypercoagulability or a proinflammatory state), or due to critical illness in patients with previously asymptomatic cerebrovascular disorders (16). We did observe a high mortality rate (30%) in patients who experienced a stroke in our study.

Seizures occurred in 2.4% of our cohort, 85% of which were new onset seizures. No cases of status epilepticus were confirmed in our cohort. Although seizures have been reported with other coronavirus infections, the evidence for association with COVID-19 remains unclear and may be related to cerebral hypoxia in some patients. A recent study of 304 patients diagnosed with COVID-19 (108 with severe infection) only documented two “seizure like” episodes, attributed to acute stress reaction and hypocalcemia, with no confirmed cases of new-onset seizures (17). Seizures were reported in six patients (0.7%) in the ALBACOVID registry, mostly in severe stages of disease. Of these, only one had a previous diagnosis of epilepsy. None of the cases were complicated by status epilepticus (12). Overall, our cohort and other recent studies do not support an additional risk of symptomatic seizures or status epilepticus in patients with COVID-19, although sub-clinical seizures may be under- recorded, especially in patients with stupor and coma.

We observed an overall mortality rate of 20.9%. One hundred fourteen of our hospitalized patients came from extended care facilities (ECF), including skilled nursing facilities and nursing homes. In this subset of ECF patients alone, the mortality rate was 32.5%, significantly higher than patients not presenting from an ECF (18%; *p* = 0.002). ECFs are believed to be high-risk settings, possessing a multitude of intrinsic risk factors allowing for ease of transmission of infectious diseases amongst residents. These risk factors include having an elderly population abiding within close quarters, with many, if not all of them, diagnosed with chronic diseases. Several studies in the United States, as well as in Europe and Asia, have further looked into the vulnerability of this patient population during this pandemic. This was first observed in the US in late March, when 101 ECF residents were confirmed to have COVID-19, all linked to one ECF in King County, Washington, with a respective mortality rate of 33.7% (18).

We were curious as to how the presence of underlying neurological disease, whether designated as a major disease vs. a minor disease, impacted COVID-19 disease progression and outcomes. In our study, we found that although patients with HND were more likely to die, HND was not an independent predictor of death. Du et al. identified history of cerebrovascular diseases as a predictor of mortality in COVID-19 patients (1). Twelve percent of patients in our cohort had history of cerebrovascular diseases, mortality rate in this group was 29.6% compared to patients without a history of cerebrovascular disease (19.7%; *p* = 0.06).

## Limitations

Our study has many limitations, mainly derived from its retrospective design. Treatment protocols, utilization of neuroimaging and electroencephalogram, neurology team consultations, and thorough neurological assessments were variable among this cohort and during the study period. Diagnosis of encephalopathy was not confirmed by a neurologist in most cases, although neurology teams were involved in the care of all patients with ischemic strokes and seizures. Our outcomes were recorded at discharge as long-term outcomes were not available at this time. Lastly, clinical severity and mortality were high due to the large number of nursing home patients in this cohort.

## Conclusions

In our cohort of hospitalized COVID-19 patients, a major neurological manifestation during disease course was an independent predictor of mortality. Additionally, having a pre-existing underlying neurological disease did not independently influence the outcome of COVID-19. Given the limitations of this retrospective study, we view these findings as a preliminary hypothesis generating framework for larger studies to investigate the impact of major neurological manifestations in hospitalized COVID-19 patients.

## Data Availability Statement

The datasets presented in this article are not readily available because all data generated for this study is contained within the manuscript. Requests to access the datasets should be directed to Mouhammad.Jumaa@utoledo.edu.

## Ethics Statement

The studies involving human participants were reviewed and approved by University of Toledo and ProMedica Toledo Hospital. Written informed consent from the participants' legal guardian/next of kin was not required to participate in this study in accordance with the national legislation and the institutional requirements.

## Author Contributions

HS, EA, and MJ designed and conceptualized the study. HS, EA, IS, SL, GD, JD, NK, KG, MA-C, SP, RA, and FS played a major role in data acquisition. HS and MJ analyzed and interpreted the data. All authors revised the manuscript for intellectual content.

## Conflict of Interest

The authors declare that the research was conducted in the absence of any commercial or financial relationships that could be construed as a potential conflict of interest.
