# Supplementary material for: Neurological Predictors of Clinical Outcomes in Hospitalized Patients With COVID-19
Source: Front Neurol. 2020 Oct 30;11:585944. doi: 10.3389/fneur.2020.585944 (PMC7662675; doi:10.3389/fneur.2020.585944)
Supplement: Supplementary file 1 [file Data_Sheet_1.docx]

Supplemental Figure 1. Death Rate Distribution across Age Groups.

Supplemental Table 1: Non-Neurologic Presenting Symptoms.

|  | Participants n=574 (%) |
| --- | --- |
| Presenting Symptoms |  |
| SOB / Hypoxia | 458 (79.8) |
| Fever | 370 (64.5) |
| Cough | 400 (69.7) |
| Fatigue | 261 (45.5) |
| Diarrhea | 160 (27.9) |
| Chest Pain | 101 (17.6) |
| Abdominal Pain | 62 (10.8) |
| Throat pain | 30 (5.2) |
| GI Bleeding | 10 (1.7) |
| Heart Failure | 10 (1.7) |
| Syncope | 9 (1.6) |
| * SOB: shortness of breath |  |

Supplemental Table 2: Laboratory Values on Presentation.

|  | Mean (SD) | Median (Range) |
| --- | --- | --- |
| Laboratory Results |  |  |
| WBC Count | 7.7 (4.6) | 6.5 (5-8.9) |
| Absolute Lymphocyte Count | 1.3 (2.6) | 0.9 (0.7-1.3) |
| Absolute Neutrophil Count | 6.0 (5.3) | 4.7 (3.4-7.1) |
| ALT | 36.07 (45.5) | 25 (16-38) |
| AST | 50.7 (65.6) | 34 (26-53) |
| Bilirubin | 0.7 (0.8) | 0.6 (0.4-0.9) |
| BNP | 209.5 (448.1) | 62 (29.3-183.5) |
| BUN | 28.3 (26.4) | 62 (29.3-183.5) |
| CK | 548.5 (1585.7) | 152.5 (68.3-388.3) |
| CO2 | 29.6 (11.0) | 26 (23-35) |
| Creatinine | 2.3 (7.6) | 1.1 (0.8-1.6) |
| CRP | 42.6 (155.7) | 11.1 (5.4-21.3) |
| D-dimer | 1021.5 (3889.1) | 314.5 (150-583.5) |
| ESR | 57.6 (30.2) | 56 (35-76.5) |
| Ferritin | 772.2 (961.7) | 470 (219-894) |
| Fibrinogen | 588.5 (243.8) | 607 (462-742) |
| INR | 1.7 (4.7) | 1.2 (1.1-1.3) |
| LDH | 352.4 (406.0) | 290 (221-387) |
| Platelets | 222.4 (104.4) | 203 (162-262) |
| Procalcitonin | 3.4 (37.0) | 0.1 (0.05–0.46) |
| Prothrombin Time | 17.5 (33.1) | 13.8 (12.88–15.2) |
| Partial Thromboplastin Time | 35.9 (39.2) | 30.8 (28-34.15) |
| Troponin | 0.1 (0.7) | 0.02 (0.01 – 0.05) |
| * WBC: white blood cell; ALT: alanine aminotransferase; AST: aspartate aminotransferase; BNP: brain natriuretic peptide; BUN: blood urea nitrogen; CK: creatine kinase; CO2: carbon dioxide; CRP: C-reactive protein; ESR: erythrocyte sedimentation rate; INR: international normalized ratio; LDH: lactate dehydrogenase | | |

Supplemental Table 3: Hospital Course, Disposition, and Re-admission Rates of COVID-19 Cohort.

|  | Participants, n=574 (%) |
| --- | --- |
| Hospital Course |  |
| Acute kidney injury | 226 (39.4) |
| Arrhythmia | 114 (19.9) |
| ARDS | 100 (17.4) |
| Cardiac injury | 93 (16.2) |
| Sepsis | 93 (16.2) |
| Septic shock | 67 (11.7) |
| GI Bleeding | 17 (3.0) |
| Pulmonary embolism | 13 (2.3) |
| Skin rash | 11 (1.9) |
| DVT | 8 (1.4) |
| Ischemic toes | 3 (0.5) |
| ICU Care | **175 (30.5)** |
| Vasopressors | 120 (20.9) |
| Mechanical ventilation | 127 (22.1) |
| Non-invasive PPV | 30 (5.2) |
| Prone positioning | 282 (49.1) |
| Dead or hospice | **120 (20.9)** |
| Dead | 99 (17.3) |
| Hospice | 21 (3.7) |
| Disposition | **454 (79.1)** |
| Home | 326 (56.8) |
| Rehabilitation Facility | 5 (0.9) |
| Skilled Nursing Facility | 123 (21.4) |
| Re-admission | **46 (8.0)** |
| Worsening symptoms | 16 (2.8) |
| Disease complication | 17 (3.0) |
| Neurological complication | 1 (0.2) |
| Other | 13 (2.3) |
| *ARDS: Acute respiratory distress syndrome; GI Bleeding: gastrointestinal bleeding; DVT: deep venous thrombosis; ICU: intensive care unit; PPV: positive pressure ventilation | |

Supplemental Table 4: Treatment of Hospitalized Patients with COVID-19

|  | Participants, n=574 (%) |
| --- | --- |
| Treatment |  |
| Anticoagulation | **70 (12.2)** |
| DOAC* | 61 (10.6) |
| Warfarin | 9 (1.6) |
| Antiviral | 13 (2.3) |
| Antibiotics | 403 (70.2) |
| Antiplatelets | 157 (27.4) |
| Convalescent Plasma | 4 (0.7) |
| Continuous renal replacement | 20 (3.5) |
| Hydroxychloroquine | 334 (58.2) |
| Immunoglobulins | 4 (0.7) |
| Stem cells | 7 (1.2) |
| Steroids | 108 (18.8) |
| Tocilizumab | 4 (0.7) |
| *DOAC: Direct acting oral anticoagulants |  |

Supplemental Table 5: Baseline, Presentation, and Treatment Data in COVID-19 patients with and without neurological symptoms.

|  | **No Neurological Symptoms**  **n=192 (%)** | **Neurological Symptoms**  **n=382 (%)** | P-value |
| --- | --- | --- | --- |
| **Baseline Demographics** | | | |
| Age | 57.8 ± 17.6 | 72.0 ± 13.2 | 0.71 |
| Female | 111 (57.8) | 187 (49) | 0.05 |
| Ethnicity |  |  |  |
| Caucasian | 107 (55.7) | 221 (57.9) | 0.64 |
| African American | 73 (38) | 145 (38) |  |
| Asian | 3 (1.6) | 2 (0.5) |  |
| Hispanic | 7 (3.6) | 12 (3.1) |  |
| Other | 2 (1) | 2 (0.5) |  |
| Healthcare worker | 14 (7.3) | 39 (10.2) | 0.29 |
| ECF resident | 38 (19.8) | 74 (19.4) | 0.91 |
| BMI | 33.6 ± 8.8 | 31.4 ± 18.9 | 0.18 |
| CAD | 34 (17.7) | 78 (20.4) | 0.65 |
| CHF | 31 (16.1) | 57 (14.9) | 0.71 |
| CKD | 32 (16.7) | 94 (24.6) | 0.03 |
| COPD | 33 (17.2) | 63 (16.5) | 0.91 |
| DM | 63 (32.8) | 160 (41.9) | 0.04 |
| Hypertension | 128 (66.7) | 275 (72) | 0.21 |
| Smoking | 70 (36.5) | 133 (34.8) | 0.71 |
| **Presentation** | | | |
| Abdominal Pain | 17 (8.9) | 43 (11.3) | 0.47 |
| Cough | 130 (67.7) | 166 (43.5) | 3.7 x 10-8 |
| Diarrhea | 46 (24) | 111 (29.1) | 0.23 |
| Fatigue | 74 (38.5) | 186 (48.7) | 0.03 |
| Fever | 108 (56.3) | 258 (67.5) | 0.01 |
| SOB / Hypoxia | 143 (74.5) | 311 (81.4) | 5.2 x 10-19 |
| Throat pain | 6 (3.1) | 24 (6.3) | 0.12 |
| Severity |  |  |  |
| Moderate | 128 (66.7) | 221 (57.9) | 0.13 |
| Severe | 42 (21.9) | 106 (27.7) |  |
| Critical | 22 (11.5) | 55 (14.4) |  |
| **Treatment & Complications** | | | |
| Days in Hospital | 8.1 ± 5.2 | 14.1 ± 11.6 | 0.06 |
| ICU admission | 43 (22.4) | 128 (33.5) | 0.007 |
| Days in ICU | 2.2 ± 5.3 | 8.3 ± 11.6 | 0.00003 |
| Days on MV | 1.6 ± 4.8 | 7 ± 10.9 | 0.001 |
| AKI | 61 (31.8) | 158 (41.4) | 0.03 |
| Antiviral | 4 (2.1) | 9 (2.4) | 1 |
| Antibiotics | 137 (71.4) | 259 (67.8) | 0.44 |
| Hydrochloroquine | 98 (51) | 232 (60.7) | 0.03 |
| Steroids | 27 (14.1) | 78 (20.4) | 0.07 |
| CRRT | 1 (0.5) | 19 (5) | 0.006 |
| PPV | 45 (23.4) | 115 (30.1) | 0.09 |
| ARDS | 19 (9.9) | 79 (20.7) | 0.001 |
| Arrhythmia | 32 (16.7) | 82 (21.5) | 0.19 |
| Cardiac injury | 24 (12.5) | 68 (17.8) | 0.12 |
| DVT | 1 (0.5) | 7 (1.8) | 0.28 |
| PE | 6 (3.1) | 6 (1.6) | 0.23 |
| Superimposed bacteremia | 23 (12) | 57 (14.9) | 0.37 |
| Sepsis | 27 (14.1) | 64 (16.8) | 0.47 |
| Septic shock | 13 (6.8) | 52 (13.6) | 0.02 |
| Vasopressors | 26 (13.5) | 92 (24.1) | 0.003 |
| Mortality | 31 (16.1) | 85 (22.3) | 0.10 |
